# Editorial Chit-Chat

**Published:** 1879-10

**Authors:** 


### Editorial Chit-Chat.

REV. THOS. K. BEECHER, D. D.—So the Illinois College, at Jacksonville, Ill., have dubbed him,—an honor most worthily bestowed.

REMOVAL.—Dr. J. B. Sargent, formerly of Tyrone, has removed to Tully, Onondaga Co. The doctor is a very capable physician, as the denizens of Tully will speedily discover.

STICKING PLASTERS.—We have heard an intimation that Herrick had offered to purchase the leather coverings to the Opera House seats, for "sticking plasters." If they will adhere to the skin as they do to a coat, or pair of pants, they will outstrip all competition.

GOING ABROAD.—Dr. S. O. Gleason and his daughter Dr. A. A. Gleason, will spend the winter in London and Paris. They will make a tour of the large hospitals, and if anything new in the method of treating the sick can be found, they will speedily take it in.

THAT BOOK.—The publishers of "BODINES" display a startling array of "notices of the press," well calculated to make our readers believe that the book is quite a success. In our advertising columns will be found two pages of these criticisms.

MR. TOWNER AND JOURNALISM.—We do not agree with Mr. Towner in all he says of the editors, actors and actresses of New York. Many of the people mentioned in his article, have been favorites with the American people for years, and justly so, we think. The article is very readable however, and gives an insight into the manner of conducting the great New York dailies, not generally known.

THAT MONUMENT.—Our energetic fellow citizen, Francis G. Hall, who made such a success of the Loan Exhibition, and of the Sullivan Centennial Celebration, now proposes to engineer a project to build a monument to Adam. Mr. Hall considers it a shame that the old gentleman's claims to monumental honors have been so long neglected. Subscription books have been opened, and the monument fund is being liberally subscribed to. Mark Twain has been engaged to deliver the oration, and a big time will be had over the erection of the first and only monument to the father of the human race.

"SHORTY" Hamilton—the renowned troutist of Lycoming creek, has lately become the father of twins. "Shorty" says that he has caught two trout at a time often, but twins never.—*Williamsport Gazette and Bulletin.*

Name one "Charles" and the other "Robert," Shorty, and we will send you a copy of BODINES.

THE BARBED FENCE.—Perhaps that contrivance called the barbed fence, may be economical and durable, cheaper than the wooden sort, but it is one of most cruel inventions. We read and hear of animals almost daily that are injured—maimed and crippled, by running against them.

We believe Mr. Bergh would have good reason to complain of these fences and seek to have their use forbidden upon the ground of needless cruelty to animals.

THE CHAIN GANG.—We have before urged the employment of city violaters of the law, upon our streets. Inasmuch as our thoroughfares are constantly in need of repairs for which laborers are hired, and as the jail always contains a dozen or more criminals who are fed at the city's expense, we see no reason why they should not be worked upon our streets to the extent at least of paying their board bills and cost of prosecution. We cannot see any injustice to anyone in such a procedure, but contrariwise, can see where it would save the city thousands of dollars. Let a chain gang be organized, and our offenders against law and good order be compelled to work. We think such a procedure would exert a healthy influence upon those recklessly inclined and put money in the public purse.

REV. W. H. H. MURRAY.—The newspapers throughout the country have behaved scandalously and ungenerously toward Mr. Murray.—He deserves better treatment at their hands. He has never proved himself other than a christian gentlemen—an honest man. He has written most charming sketches of out-door life; his story of "The man who did'nt know much" being one of the must beautiful conceptions in our language, the production of which should alone have immortalized the writer. Mr. Murray's failure in business does not argue that he is a rogue, unworthy the sympathy of his fellow men; yet have the journals so regarded him. Thousands of men have made worse failures, and have escaped this wholesale denunciation and blackguarding. Nothing has appeared showing Mr. Murray to have been anything but unfortunate in his business transactions,—misfortunes too, from which he could have extricated himself but for the meddlesome interference and slander of the press. We never knew of a more unjust and undeserved fate than that visited upon this gentleman by the newspapers of the country.

---

SARAH BERNHART.—The whims of people, evolved from fashion, will afford us a deal of amusement if we will but look on without absorbing any portion of the fashionable contagion that happens to be uppermost.

A case in point, is that beautiful and talented actress, Sarah Bernhart. One would suppose that it would be utterly impossible to obtain permission to introduce a lewd or unchaste woman to the nobility of England. But it has become *fashionable* to rave about this beautiful courtizan, with several illegitimate children, so the dear public take her in,—children and all. We protest againt this recognition of unchastity, by respectable people.—Such women should never be permitted to enter the parlors where are congregated our young men and women. Our young folks should not be subjected to such an insult. It may be permissable to witness the performance of such a person, upon the stage of an opera house, but never in the private parlor. We hope the American ladies will not degrade their sex by giving Sarah Bernhart the entree of their dwellings when she visits this country.

---

DICK IS SATISFIED.—Dick is a most excellent printer and presides over the job printing house from which the BISTOURY is issued. He takes especial delight (as we believe do all printers) in calling for "copy" just as the editor has sent in his last article that he was promised would fill the very last column.

It was therefore with a grim smile of satisfaction that we met Dick upon the veranda this morning and heard his troubled exclamation: "Why, Doctor, I have enough matter set up now to fill the BISTOURY chock-full and we havn't reached the editorial yet! What in thunder shall I do?"

Dick seemed perplexed and unhappy over the influx of copy. He would have known just what to have done had there been too little of it, but a surfeit, was an unusual experience, and puzzled him. At last, after much engineering, we concluded that the editorial department had better suffer than to have any of our excellent contributions lay over until next issue; accordingly, we cut our article on over-education smack in two—in the middle, and withheld a capital essay on autumn-gypsying for all of which our readers should be duly thankful.

---

"IRREGULAR" CONSULTATIONS.—Our city medical professors are much given to instructing the young physician never to consult with a homeopathic or other physician not a member of some "regular" medical society. We observe that these same professors never obey their own instructions in this respect, but habitually and invariably go at the call of any physician,—no matter what his pathy may be, or his standing even, in the community—and perform surgical operations, or give medical advice without once inquiring about the physician by whom they are summoned, if only the fee is secure.

We have known these same distinguished gentlemen to come to the country and do surgical operations for a fee less than one-half the sum asked by a fellow surgeon, not a resident of the great metropolis. Yet are these professors great sticklers—in their lectures to the students—for professional etiquette, or the upholding of the code of ethics.

Indeed, we believe that nowhere and by no class of men is the code more outrageously violated than in our Medical Colleges and by the Professors themselves. They do not hesitate to consult with and operate for irregular physicians; they will undercharge the country surgeon to secure his case; and they advertise themselves into notoriety in their catalogues, medical essays, and reports of meetings of societies in a manner that would cause a modest practitioner to blush.

Now, if it is right for a professor in a medical college to consult with an irregular practicioner, we can not see the sin in regular and irregular physicians of the same town consulting together.

If the medical gentlemen of New York and Philadelphia expect physicians of the country to obey the code of ethics, they should themselves adhere to its instructions. This they have never done, nor will they ever.

